# Inhibitory Activity of *Avicennia marina*, a Medicinal Plant in Persian Folk Medicine, against HIV and HSV

**Published:** 2013

**Authors:** Rahele Namazi, Rezvan Zabihollahi, Mandana Behbahani, Abbas Rezaei

**Affiliations:** a *Department of Pharmaceutical Biotechnology, School of Pharmacy, Shahid Beheshti University of Medical Sciences, Tehran, Iran. *; b *Hepatitis and AIDS Department, Pasteur Institute of Iran, Tehran, Iran. *; c *Medical School, Isfahan Medical Sciences University, Isfahan, Iran. *; d *Students Research Committee, Shahid Beheshti University of Medical Sciences, Tehran, Iran.*; e *Department of Microbial Biotechnology, Faculty of Advanced Sciences and Technologies, University of Isfahan, Isfahan, Iran.*

**Keywords:** *Avicennia marina*, Herpes simplex virus, Human immunodeficiency virus, Antiviral activity, Medicinal plant

## Abstract

*Avicennia marina *(Avicenniaceae) is a species of mangrove tree used for treatment of small pox lesions in Persian folk medicine. The antiviral activity of methanol, ethanol, water, chloroform and *n*-hexane extracts was evaluated against HIV-1 and HSV. Methanol extract had the highest antiviral activity and the most polar fraction of this extract (fraction D) inhibited HSV with TI and SI values of 57.1 and 133; however, it showed mild activity against HIV with SI value of 6.25 (fraction 3). The anti-HSV activity of active fraction was confirmed using FLASH-PCR. Phytochemical investigation revealed that fraction D encompasses flavonoids compounds. The time-of-addition study demonstrated that fraction D disturbs viral replication after penetrating to the cell. *A. marina *was endowed with fragments by which found to be able to inhibit replication of HSV after entry but did not show significant potency against HIV-1. This promotes further investigation in anti-HSV drug discovery.

## Introduction

Herpes simplex virus (HSV) is an enveloped one with a single large double stranded DNA (1). HSV is a member of the Alphaherpesvirus subfamily called Herpesviridae which is widely distributed in general population (2). HSV is a common pathogen which can elicit multiple disease states including cold sores, gingivostomatitis, keratoconjuctivitis, genital infections, chicken pox, shingles, encephalitis, mononucleosis, congenital defects, some B-cell lymphomas and sarcomas (3). HSV tends to persist in ganglia neurons after primary infection (4). Regular or sporadic reactivations of the latent viral genome may result in lifetime herpetic relapse (3). There are nucleoside analog compounds which are approved for treatment of herpes infection (5, 6). These agents inhibit viral DNA synthesis after the activation by HSV thymidine kinase (7). Extensive clinical use of these drugs has led to the formation of resistant viral strains (8, 9).

 The infectious cause of acquired immunodeficiency syndrome (AIDS) is human immunodeficiency virus (HIV) (10). AIDS is characterized by the depletion in CD4-positive T lymphocytes count. Despite considerable success of highly activating antiretroviral therapy (HAART), AIDS has remained one of the most important health problems worldwide (11, 12). Emergence of resistant strains formation and toxicity of current available drugs make an impetus to find new anti-HIV therapy strategies (13, 14). Medical plants are promising candidates for developing new antiviral drugs. The ethnomedicinal usage against infectious diseases could be considered for pharmacological studies (15). Mangroves are medicinal plants and extracts from different parts of them are widely used through the world (16). These are considered as rich sources of steroids, triterpenes, saponins, tannins, alkaloids and flavonoids (17, 18). Antiviral, antibacterial and antifungal activity of the extracts from these plants have been shown in former studies (16, 18). *A.* *marina *also known as white or grey mangrove is a species of mangrove tree classified in the plant family of Avicenniaceae plant family (19). This plant grows in Qeshm mangrove forests of Iran located in Persian Gulf (20). These forests mostly include two tree species of *A. marina *and *Rhizophora mucronata *(Rhizophoraceae) (21). The aerial parts of *A. marina *have traditionally been used for treating rheumatism, small pox, abscess and ulcers (16, 18). This plant has also been used in Persian folk medicine for treatment of some other infectious diseases (22). Beside conventional usage, some pharmacological activities such as anti-bacterial (23-25) and inducing apoptosis in cancer cells (26) have been reported for crude extracts from this plant. Extracts from *A. marina *have antiviral activity and are able to inhibit *hepatitis B virus *(HBV) and Encephalomyocarditis virus (EMCV) replication (27). Safety studies have shown that* A. marina *is safe for *in vivo *administration and no behavioral changes and mortality/morbidity would be experienced among objectives (28). The current study investigates the inhibitory potential of *A. marina *against HSV-1 (KOS strain) and HIV-1 (NL4-3). The leaf crude extracts were obtained using different solvents and assayed for antiviral and cytotoxicity. The most active extract was fractionated to determine the active portion. The chemical nature of active fraction was studied using phytochemical examinations. The mechanism of action was investigated using time-of-addition study.

## Experimental

*Plant material and extraction*

*A. marina *leaves were collected from mangrove forests (Qeshm Island, Persian Gulf, Iran), in April 2009. The authenticity of plant samples were identified and confirmed by anatomical and morphological techniques by our co-author, M. Behbahani, Ph.D. (Faculty of Advanced Sciences and Technologies, University of Isfahan, Isfahan, Iran). Voucher specimens of plant materials were deposited as the reference (Sample 11352) in Pasteur institute of Iran, Tehran, Iran. The leaves were carefully cleaned, shade dried and powdered. The powdered material was stored in a closed air-tight plastic container at low temperature. The powdered plant material (50 g) was extracted with 300 mL of each solvent (methanol, ethanol, water, chloroform and *n*-hexane) by maceration (3×24 h) at room temperature. The collected solvents were concentrated by rotary vacuum evaporator (Stero glass, Italy) at 45°C and then dried using a freeze dryer (Zirbus, Germany) (29). All extracts and acyclovir (extracted from commercial tablet) were dissolved in dimethyl sulphoxide (DMSO). The final concentration of DMSO was 0.1% v/v in cell culture environment.

*Fractionation procedures*

Dried methanol extract (2.5 g) was dissolved in 5 mL of methanol 98% (Merck, Germany) and fractionated using column chromatography (30). The silica gel column that contained *n*-hexane extract was eluted with *n*-hexane and acetone (8:2 to 2:8 v/v) followed by adding 100% methanol to yield ten fractions. Eluted fraction 10 (12% w/w yield) was further subjected to column chromatography on silica gel and eluted with chloroform and methanol (8:2 to 4:6 v/v) to harvest fractions A, B, C and D with yield of 23, 25, 27, 22% w/w, respectively.

*Phytochemical evaluation*

Phytochemical study was performed as previously described (31, 32). Fraction D (200 μg) was dissolved in dilute hydrochloric acid and subjected to alkaloids presence analysis (Mayer’s and Dragendroff’s tests). Mayer’s test was conducted by treating fraction D solution with Mayer’s reagent (Potassium Mercuric iodide). Formation of a yellow creamy precipitate is indicative for alkaloids. Total alkaloid content of fraction D was determined using Dragendorff precipitation assay. Fraction D solution was treated with Dragendroff’s reagent (solution of potassium bismuth iodide). The formation of red precipitate indicates the presence of alkaloids.

 To determine the presence of flavonoids, few drops of sodium hydroxide solution were added to fraction D solution (Alkaline Reagent test). Formation of intense yellow color which becomes colorless after addition of HCl denotes the presence of flavonoids. To perform the zinc hydrochloric acid reduction test, a small amount of Zinc dust (0.5 g) and concentrated HCl (two drops) were added to ethanol solution of fraction D. Appearance of red-orange color after a few minutes demonstrates the presence of flavonoids.

 Total tannins content was determined by Gelatin Test. Gelatin solution (1% v/v) containing NaCl was added to the fraction D. Generally, formation of white precipitate confirms the presence of tannins.

*Cell culture*

African green monkey kidney cells (Vero) and human ecmbryonic kidney cells (HEK293) were obtained from Pasteur institute of Iran (Tehran, Iran). Cells were cultured in Dulbecco’s Modified Eagle Medium (DMEM, Gibco, USA) supplemented with 10% heat-inactivated fetal bovine serum (FBS, Gibco, USA), 100 U/mL penicillin, 100 μg/mL streptomycin, 0.33 g/L l-Glutamine (Gibco, USA) and 1mM sodium pyruvate (Gibco, USA) in a CO_2_ incubator.

*Viruses*

Pseudotyped SCR HIV-1 virions with *vesicular stomatitis virus *surface glycoprotein (VSVG) were prepared using transfection (33, 34). Plasmids were transfected into HEK293T cells using Polyfect transfection reagent (Qiagen, USA) according to the user manual. Briefly, 3.7 × 10^5 ^HEK293T cells were seeded in 6-wells plate and transfected after 24 h. HEPES at the concentration of 25 nM was added to cells environment during transfection. Plasmid mixture (pSPAX2, pMD2G and pmzNL4-3) was used for each well and transfection complex was removed after 7 h (34, 35). Virus containing supernatants were harvested at 24, 48 and 72 h post transfection, pooled and stored at 4ºC. Pooled supernatant was clarified after 5min centrifuging at 1.5×10^4^ g and filtering thought 0.22 μm filters. Viruses were stored at -70ºC and determined for infectious titer (36, 37). KOS strain of HSV-1 was used in this study. To prepare the virus stock, 4×10^5^ Vero cells were seeded in the 6-wells plates and infected after 24 h. The supernatants that contained virions were harvested each day after infection until day four. The harvested supernatants were pooled and clarified using 0.22 μm filters. Virus stock was stored at - 70ºC and tittered for plaque forming activity.

*Plaque reduction assay*

The anti-HSV activity was investigated using plaque reduction assay as previously described with minor modifications (38). Vero cells were seeded in 24-wells culture plate containing complete DMEM supplemented with 5% FBS (Falcon, USA) at a density of 3.5×10^5^ cells per well. The cells were cultured for 24h to reach at least 98% of confluence. The cells monolayer was infected with 40 PFU (plaque forming unit) of HSV-1 and incubated to adsorb virions for 1h. The infected cells were washed and overlaid with 1.2% methylcellulose supplemented medium. DMSO (%0.1v/v) and 5mg/ml acyclovir were used as negative and positive controls. After 3 days, the overlay medium was removed and the cell monolayer was washed and fixed with DMEM and methanol. The number of plaques was counted after staining with %0.5 crystal violet. The antiviral activity was determined by the following formula: 

Toxicity percentage = $1-\frac{\mathrm{OD test}}{\mathrm{OD control}}\times 100$

The minimal concentration required to decrease the number of plaques by 50% (IC_50_) was determined by regression analysis of the dose response curve generated from the data (39). 

*HIV replication assay*

Anti-HIV-1 activity was measured using single cycle replication assay. VSV-G pseudotyped single cycle replicable (SCR) HIV- 1 virions were used to infect HEK293T cells in the presence of extracts (34). The cells were placed into the 96-wells plate at the confluence of 6×10^3^ cells per well and incubated for 24h. SCR HIV virions (600 ng P24/well) were then added into the wells and underwent incubation for 20h at 37°C. The cells were washed two times with prewarmed DMEM medium to remove any unbound virion and fed with 200μl of fresh medium. Plates were incubated for additional 48h to let the virions replicate for one cycle. P24 load of cells supernatant was analyzed after centrifuging plates at 3×10^3^g for 10min. P24 capture ELISA (Biomerieux, France) was used to evaluate the P24 load of the cells supernatant.

Nevirapine (100nmol) and DMSO (1%) were used as positive and negative control. The minimal concentration required to suppress the load of P24 by 50% (IC_50_) was determined by regression analysis of the dose response curve generated from the data.

*FLASH-PCR*

FLASH–PCR was performed to confirm the anti-HSV-1 activity of fraction D. Vero cells were cultured in 24-wells (10^5^/well) culture plate (Falcon, USA). After 24 h, cells were infected with 100PFU of HSV-1 for 1h and unbound virions were washed out. Fraction D concentration was kept constant from the time of addition to the end of the test. Acyclovir was considered as the positive control. Later, treated cells were harvested and prepared for DNA isolation. Viral DNA was isolated using “High Pure Viral Nucleic Acid Kit” (Roche, Germany) according to the manufacturer’s instructions. FLASH-PCR was performed using “Herpes simplex virus 1, 2 detection FLASH-PCR kit” (Bioron, Germany) according to user manual. The results of PCR amplification were analyzed in FLASH format by measuring the fluorescence on a Gene detector

(DNK-Tekhnologiya, Russia).

*Time-of-addition study*

The time-of-addition inhibitory effect of fraction D was examined by plaque reduction assay according to previously described procedures with minor modifications (38). Briefly, monolayer of the cells in 24-wells plate were infected by 40PFU of HSV for one hour and then overlaid with methylcellulose medium. Fraction D (20 μg/ mL) was added to the wells before (- 2 and – 4 h), during (0 h) and after infection (2 and 4 h). To add the fraction during infection the virus and fraction were simultaneously added. The cells’ monolayers were washed so that the fractions having been added before infection were removed but the concentration of the added fraction during infection was kept unchanged. After three days, the wells were washed and stained after fixation as mentioned above.

*Cytotoxicity assay*

The cytotoxic effect of *A. marina *crude extracts and fractions on Vero cells was measured by XTT (sodium 3_-[1 (phenylaminocarbonyl)- 3,4-tetrazolium]-bis(4-methoxy-6-nitro)benzene sulfonic acid) Proliferation assay kit (Roche, Germany) according to the instruction (40, 41). Vero cells were seeded in 96-wells culture plate (10^4^ cells/well) (Falcon, USA) containing 200 μL of phenol red free medium. After 72 h, the cells medium was replaced just before the assay.

Plates were incubated 4h in a CO_2_ incubator after addition of the XTT solution to the wells. The test and reference optical densities (OD) were measured using an ELISA reader (stat fax2100, Awareness, USA), at 450nm and 630 nm, respectively. The cytotoxic effect was calculated by the following formula:

Toxicity percentage = $1-\frac{\mathrm{OD test}}{\mathrm{OD control}}\times 100$

*Statistical analysis*

All experiences were done in triplicate and two independent experiences. Results are expressed as the mean ± SD and the IC_50_ (50% inhibitory concentration) IC_25_, CC_50_ (50% cytotoxic concentration) and CC_25_ values were calculated by analyzing the dose response curve. One way anova was used to calculate P values for means between control and tested samples. A p-value of less than 0.005 was considered to be statistically significant. 

## Results and Discussion

*Antiviral potential of the A. marina crude extracts*

In this study, *A. marina *leaf crude extracts were investigated for anti-HSV-1 (KOS) and HIV-1 (NL4-3) activity as well as cellular toxicity. The final yield of methanol, ethanol, aqueous, chloroform and *n*-hexane crude extracts mass were 10.2, 8.5, 2.8, 3.5 and 5.6 g, respectively. The therapeutic index (TI) value of methanol, ethanol, aqueous, chloroform and *n*-hexane extracts for inhibition of the HSV replication were 2.5, 1.04, 1.0 , 0.61 and 0.19, respectively (Table 1). 

**Table 1:** Anti-viral potential of the *Avicennia marina *crude extracts

**Extract**	**HSV Inhibitory (KOS)**			**HIV Inhibitory (NL4-3)**			**Cytotoxicity**
	**IC** ^ a^_ 50_	**TI** ^b^	**SI** ^c^	**IC**_50_	**TI**	**SI**	**CC** ^ d^_50_
Methanolic	80	> 2.5	8.67	76	> 2.6	5.2	> 200
Ethanolic	192	1.04	0.17	188	1.06	0.22	200
Aqueous	> 200	1	1.21	195	> 1.02	1.94	> 200
Chloroformic	180	0.61	0.001	132	0.83	0.003	110
n-hexanic	176	0.19	0.007	45	0.77	0.2	35

Note: ^a^IC_50_ concentration with 50% inhibition activity (μg/mL); ^b^TI therapeutic index; ^c^SI selectivity index; ^d^CC_50 _concentration with50% cellular toxicity (μg/mL

The selectivity index (SI) value of methanol extract for inhibiting the HSV replication was 8.67. The crude extracts inhibit HIV with TI value of 2.6, 1.06, 1.02, 0.83 and 0.73, respectively (Table 1). Methanol extract had the highest potential for inhibiting HIV with SI value of 5.2. The IC_50_ of methanol extract for HSV and HIV were 9 μg/mL and 15 μg/mL, respectively which denote the notable potency of this extract. This indicates that methanol extract might contain antiviral active compounds. As cytotoxicity of crude extracts was determined by XTT assay (Table 1), the CC_50_ of methanol extract was > 200 μg/mL showing minor toxicity. It showed that methanol extract might contain lesser toxic components. As it is evident in Table 1, crude methanol extract was the most potent one among all with the highest TI and SI values against both anti-HIV and HSV. Regarding these data, methanol extract was targeted for fractionation procedure to specify active antiviral compounds.

*Antiviral potential of the fractions*

Ten fractions were yielded from the methanol crude extract after fractionation on the silica gel column. The inhibitory activities of these fractions against HSV-1 and HIV-1 in addition to their cytotoxicity are shown in Table 2.

**Table 2:** Anti-viral potential of fractions from the methanol extract

**Fractions**	**Anti-HIV-1 activity**			**Anti-HSV-1 activity**			**Cytotoxicity**
	**IC ** ^a^_50_	**TI** ^b^	**SI** ^c^	**IC**_50_	**TI**	**SI**	**CC ** ^d ^_50_
1	200	> 1	1.94	200	> 1	> 1.4	> 200
2	142	> 1.4	2.12	100	> 2	3.7	> 200
3	87	> 2.29	6.25	98	> 2	4.17	> 200
4	54	> 3.7	2	82	> 2.4	2	> 200
5	63	> 3.17	2.5	76	> 2.63	7.5	> 200
6	> 200	1	1.29	175	> 1.14	2.22	> 200
7	> 200	1	1.19	200	> 1	2.63	> 200
8	> 200	1	0.99	120	> 1.66	5.57	> 200
9	195	> 1	1	15	> 13.3	12.5	> 200
10	167	> 1.19	1.39	32	> 6.25	20*	> 200
A	> 200	< 1	1	89	2.24	3.75	200
B	> 200	1	1.7	95	> 1	3.89	> 200
C	> 200	1	1	31	6.45	9.09	> 200
D	> 200	2.32	1.16	3.5	> 57.1*	133*	> 200

Note: ^a^IC_50_ concentration with 50% inhibition activity (μg/mL); ^b^TI therapeutic index; ^c^SI selectivity index; ^d^CC_50 _concentration with 50% cellular toxicity (μg/mL); *Significant difference between test sample and solvent control (p < 0.005

 Based on obtained data, fractions 1, 2, 6, 7 and 8 were not toxic and their CC_50_ was more than 200 μg/ mL. The fraction 4, 5, 9 and 10 were the most effective ones against HSV-1. Fractions 9 and 10 were lesser toxic and were significantly potent for HSV inhibition with TI value of 13.3 and 6.25, respectively. The SI value of fraction 10 was 20 for inhibiting HSV. Fractions 3, 4 and 5 were active for inhibiting HIV replication. The SI and TI values of fraction 3 were 6.25 and 2.29 for inhibiting HIV virions which imply considerable antiretroviral activity. This fraction exhibited moderate anti-HSV potential (SI of 4.17) and mild toxicity with CC_50_ of 200 μg/mL. The fractions 4 and 5 were more toxic with CC_50_ value of 2 μg/mL and 15 μg/mL, respectively. Fraction 5 also showed anti-HSV activity with IC_50_ of 2 μg/mL (SI value of 7.5). 

Regarding high TI and SI values of fraction 10 against HSV-1, it was subjected to the additional fractionation procedure. Four fractions (A-D) were harvested after secondary fractionation (Table 2). The IC_50_ and IC_25_ values of fraction D for inhibiting HSV were 3.5 and 0.75 μg/ mL, respectively. This revealed that fraction D contained highly active anti-HSV compounds which were active at low concentrations. The calculated TI and SI values for fraction D were 57.1 and 133 denoting its high anti-HSV-1 potential. The ability of fraction D for inhibiting HIV-1 is also shown in Table 2. Fraction D didn’t demonstrate any significant antiretroviral potency and this would emphasize that fraction D acts as an HSV-1 replication inhibitor in a specific manner.

*Effect of addition time on the anti-HSV-1 activity of the fraction D*

**Figure 1:**
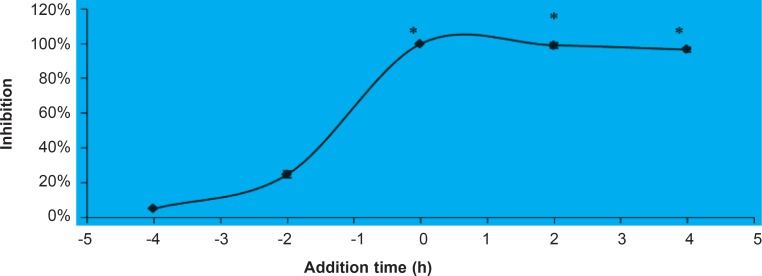
Anti-viral Activity of fraction D at Different Times of Addition. Fraction D (20 μg/mL) was added at either before (- 4 and – 2 h) or after (0, 2 and 4 h) virus infection. The extracts that were added before virus infection were rinsed off after virus exposure. The result showed that the presence of fraction D before infection cannot inhibit virus infection, although it could inhibit virus replication if be present after virus infection. *Significant difference between test sample and solvent control (p < 0.005

Infection-compound addition time course study was performed to investigate the inhibitory effect of fraction D on different stages of HSV- 1 infection. Fraction D (20 μg/mL) was added at different periods (before, during and after) of HSV-1 infection. The added fractions before infection were removed through rinsing but the concentration of fraction in other intervals (0, 2 and 4 h) was kept constant. The results showed that fraction D suppressed HSV-1 infection when added after virus inoculation (0, 2 and 4 h). The inhibitory rate was higher than 95% (Figure 1) when the fraction was added in mentioned intervals. However, the inhibitory rate declined to 25% or less when added prior to the infection (- 4 and - 2 h). The presence of fraction D before infection was not able to significantly inhibit viral replication. However, the HSV replication was almost inhibited in the presence of this fraction after viral penetration (Figure 1). This finding indicates that, although this agent could not inhibit virus adhesion and entry but efficiently blocks virus replication and multiplication cycle.

*FLASH-PCR*

**Figure 2:**
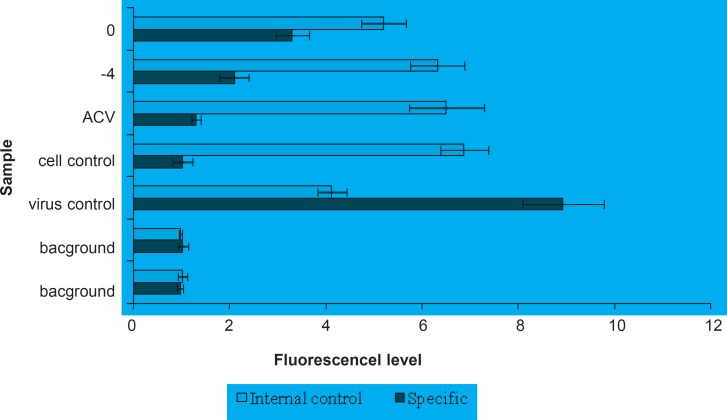
FLASH-PCR assay for anti-HSV activity of fraction D. Addition of fraction D to the cell environment significantly inhibits HSV-1 (KOS) DNA reproduction (- 4, 0) over virus control. Addition of this agent before infection (- 4) enhances its antiviral activity. Acyclovir (ACV) 5 mg/mL was used as anti-HSV control in this assay

The anti-HSV-1 activity of fraction D was confirmed using FLASH-PCR. Fraction D (20 μg/mL) was added to the cells environment at the time of infection and 4 h before that. When this fraction accompanied with HSV-1 was concurrently being added, 63.1% of virus infection was inhibited (Figure 2). However, the inhibitory rate increased to 73.6% when added at 4h before infection. As it is shown in Figure 2, the presence of fraction D significantly inhibited HSV-1 DNA multiplication. Fraction D addition before infection enhanced the anti-HSV-1 activity of this agent. These results indicated that the anti-HSV-1 activity of this fraction is detectable even at viral nucleic acid level.

*Phytochemical examinations*

Fraction D (the anti-HSV-1 active portion of *A. marina *extract) was implemented for phytochemical studies. Mayer’s and Dragendroff’s tests were negative and this indicates that fraction D does not contain alkaloid agents. Also no white precipitate was seen in the Gelatin Test (tannins presence test). The yellow color was created within alkaline reagent test and the solution became colorless after addition of HCl. This result showed that fraction D encompasses flavonoid compounds. The mentioned result was confirmed using zinc hydrochloric acid reduction test. These results indicate that fraction D may contain some flavonoid compounds. It has previously been shown that the *A. marina *is enriched with flavonoid derivate compounds (22, 26, 39, 42- 44). Anti-HSV activity of structures related to flavonoid compounds has also been reported in some previous studies (45, 46). Our results emphasize that *A. marina *leaves contain highly potent anti-HSV-1 compounds that are probably flavonoid derivates.

## Conclusion

Herein, we examined the antiviral activity of extracts from a medicinal plant named *A. marina*. This plant grows in Hara forest of Persian Gulf and is used in Persian folk medicine. The anti- HIV and HSV activity of methanol, ethanol, water, chloroform and *n*-hexane extracts and fractions were studied as well as cytotoxicity. The methanol extract showed higher antiviral activity so it was chosen for fractionation. The most polar fraction (D) of methanol extract showed significant anti-HSV activity but was not active against HIV virions. This fraction inhibits the HSV replication after entry of virions to the target cells. The anti- HSV activity of fraction D was also confirmed using FLASH-PCR method at the level of viral nucleic acids. The phytochemical examinations have shown that fraction D contains flavonoid compounds. This demonstrates that *A. marina* contains active flavonoid compounds with significant potential against HSV (KOS) virions replication inhibition after penetrating the target cells. These findings merit further investigation on antiviral activity of *A.* *marina*. We are going to continue this study by purifying the flavonoid compounds from the effective extracts of *A. marina*, and also by monitoring its inhibitory effect on other DNA viruses. Further studies will focus on *in* *vivo *investigations of the anti-HSV activity of *A. marina *in mouse models. *A. marina *could be a promising anti-HSV agent for the herbal therapy against HSV infections.

## References

[1] Kieff ED, Bachenheimer SL, Roizman B (1971). Size, composition, and structure of the deoxyribonucleic acid of herpes simplex virus subtypes 1 and 2. Virolog.

[2] Roizmann B, Desrosiers RC, Fleckenstein B, Lopez CA, Minson C, Studdert MJ (1992). The family Herpesviridae: an update. Arch. virol.

[3] Whitley RJ, Kimberlin DW, Roizman B (1998). Herpes simplex viruses. Clininic. Inf. Dis.

[4] Baringer RJ, Swoveland P (1973). Recovery of herpessimplex virus from human trigeminal ganglions. N.Eng. J. Med.

[5] Fahad A, Stepher L (1996). New anti herpes virus agent. Their target and therapeutic. Pot. Drug.

[6] Cassady KA, Whitley RJ (1997). New therapeutic approaches to the alphaherpesvirus infections. Antimicrob Chemother.

[7] Fyfe JA, Keller PM, Furman PA, Miller RL, Elion GB (1978). Thymidine kinase from herpes simplex virus phosphorylates the antiviral compound, 9-(2-hydroxyethoxymethyl)guanine. Biolog Chemist.

[8] Englund JA, Zimmerman MK, Swierkosz EM, Goodman JL, Scholl DR, Balfour HH (1990). Herpes simplex virus resistant to acyclovir. Annl. Intern. Med.

[9] Bacon TH, Levin MJ, Leary JJ, Sarisky RT, Sutton D (2003). Herpes Simplex Virus Resistance to Acyclovir and Penciclovir after Two Decades of Antiviral Therapy. Clin. Microbiol Rev.

[10] Barre-Sinoussi F, Chermann JC, Rey F, Nugeyre MT, Chamaret S, Gruest J, Dauguet C, Axler-Blin C, Vezinet-Brun F, Rouzioux C, Rozenbaum W, Montagnier L (1983). Isolation of a T-lymphotropic retrovirus from a patient at risk for acquired immune deficiency syndrome (AIDS). Science.

[11] Arora DR, Gautam V, Gill PS, Mishra N (2010). Recent advances in antiretroviral therapy in HIV infection. J.Indian Med. Assoc.

[12] Wilkin TJ, Shalev N, Tieu HV, Hammer SM (2010). Advances in antiretroviral therapy. Top. HIV Med.

[13] Cohen J (2009). HIV/AIDS. Tangled patent dispute over ‘free’ drug-resistance database. Science.

[14] Obiako OR, Murktar HM, Ogoina D (2010). Antiretroviral drug resistance--implications for HIV/AIDS reduction in sub-Saharan Africa and other developing countries. Niger J. Med.

[15] Naithani R, Huma LC, Holland LE, Shukla D, McCormick DL, Mehta RG, Moriarty RM (2008). Antiviral activity of phytochemicals: a comprehensive review. Mini Rev. Med. Chem.

[16] Bandaranayake WM (1998). Traditional and medicinal uses of mangroves. Mangrov Sal. Marsh.

[17] Bandaranayake WM (1995). Survey of mangrove plants from Northern Australia for phytochemical constituents and uv-absorbing compounds. Curr. Top. Phytochem.

[18] Bandaranayake WM (2002). Bioactive compounds and chemical constituents of mangrove plants. WetlandsEcology and Management.

[19] Duke NC, Benzie JAH, Goodall JA (1998). Genetic structure and evolution of species in the mangrove genus Avicennia (Avicenniaceae) in the Indo-West Pacific. Evolution.

[20] Danehkar A (1996). Iranian mangroves forests. Envir Sci. Quar. J.

[21] Ghasemi S, Zakaria M, Abdul-Hamid H, Yusof E, Danehkar A, Rajpar MN (2010). A review of mangrove value and conservation strategy by local communities in Hormozgan province, Iran. American Science.

[22] Rezaii Y (1993). Study on pharmacognosy effect of Avicennia marina.

[23] Manhaseneh (2000). Determination of minimum inhibitory concentration (MIC) of antibacterial agents by agar dilution. Clinical Microbial Infection.

[24] Abeysinghe PD, Wanigatunge RP, Pathirana RN (2006). Evaluation of antibacterial activity of different mangrove plant extracts. Ruhuna J. Sci.

[25] Abeysinghe PD (2010). Antibacterial activity of some medicinal mangroves against antibiotic resistant pathogenic bacteria. Indian J. Pharmaceut Sci.

[26] Shraf M, El-Ansari MA, Saleh NAM (2000). New flavonoids from Avicennia marina. Fitoterapia.

[27] Premanathan M, Kathiresan K, Nakashima H (1999). Mangrove Halophytes: A source of antiviral substances. South. Pac. Stud.

[28] Ali BH, Bashir AK (1998). Toxicological studies on the leaves of Avicennia marina (mangrove) in rats. Appl.Toxicol.

[29] Fritz D, Venturi CR, Cargnin S, Schripsema J, Roehe PM, Montanha JA, von Poser GL (2007). Herpes virus inhibitory substances from Hypericum connatum Lam., a plant used in southern Brazil to treat oral lesions. J. Ethnopharmacol.

[30] Manila AS, Sujith G, Kiran S, Selvin J, Shakir C (2009). Biopotentials of Mangroves Collected from the Southwest Coast of India. Glob j Biotechnol. Biochem.

[31] Brain KR, Turner TD (1975). The practical evaluation of phytopharmaceuticals.

[32] Evans WC (1996). Trease and Evans’ Pharmacognosy.

[33] Parsaee H, Asili J, Mousavi SH, Soofi H, Emami SA, Tayarani-Najaran Z (2013). Apoptosis Induction of Salvia chorassanica Root Extract on Human Cervical Cancer Cell Line. Iranian J. Pharmceut. Res.

[34] Sadat SM, Zabihollahi R, Vahabpour R, Azadmanesh K, Javadi F, Siadat SD, Memarnejadian A, Parivar K, Khanahmad Shahreza H, Arabi Mianroodi R, Hekmat S, Aghasadeghi MR (2010). Designing and biological evaluation of single-cycle replicable HIV-1 system as a potential vaccine strategy. 20th European Congress of Clinical Microbiology and Infectious Diseases; Austria. Clinical Microbiology and Infection.

[35] Zabihollahi R, Sadat SM, Vahabpour R, Aghasadeghi MR, Memarnejadian A, Ghazanfari T, Salehi M, Rezaei A, Azadmanesh K (2011). Development of singlecycle replicable human immunodeficiency virus 1 mutants. Acta. Virol.

[36] Rezaei A, Zabihollahi R, Salehi M, Moghim S, Tamizifar H, Yazdanpanahi N, Amini G (2007). Designing a non-virulent HIV-1 strain: potential implications for vaccine and experimental research. J. Res. Med. Sci.

[37] Svarovskaia ES, Barr R, Zhang X, Pais GC, Marchand C, Pommier Y, Burke TR, Jr, Pathak VK (2004). Azidocontaining diketo acid derivatives inhibit human immunodeficiency virus type 1 integrase in vivo and influence the frequency of deletions at two-longterminal- repeat-circle junctions. J. Virol.

[38] Cavrois M, Neidleman J, Yonemoto W, Fenard D, Greene WC (2004). HIV-1 virion fusion assay: uncoating not required and no effect of Nef on fusion. Virology.

[39] Yang CM, Cheng HY, Lin TC, Chiang LC, Lin CC (2005). Acetone, ethanol and methanol extracts of Phyllanthus urinaria inhibit HSV-2 infection in-vitro. Antiviral Res.

[40] Cheng HY, Lin CC, Lin TC (2002). Antiherpes simplex virus type 2 activity of casuarinin from the bark of Terminalia arjuna Linn. Antiviral Res.

[41] Scudiero DA, Shoemaker RH, Paull KD, Monks A, Tiemey S, Nofziger TH, Currens MJ, Seniff D, Boyd MR (1998). Evaluation of a soluble tetrazolium/ formazan assay for cell growth and drug sensitivity in culture using human and other tumor cell lines. Canc.Res.

[42] Lin CC, Cheng HY, Yang CM, Lin TC (2002). Antioxidant and antiviral activities of the Euphorbia thymifolia Linn. Biomed Sci.

[43] Rui J, YueWei G, HuiXin H (2004). Studies on the chemical constituents from leaves of Avicennia marina. Chin J.Nat. Med.

[44] Jia R, Guo YW, Hou HX (2004). Studies on the chemical constituents from leaves of Avicennia marina. Chin J.Nat. Med.

[45] Feng Y, Li XM, Wang BG (2007). Chemical constituents in aerial parts of mangrove plant Avicennia marina. Chin. Trad. Herb. Drug.

[46] Amoros M, Simoes CMO, Girre L (1992). Synergistic effect of flavones and flavonols against herpes simplex type 1 in cell culture. Comparison whit the antiviral activity of propolis. J. Nat. Prod.

[47] Lyu SY, Rhim JY, Park WB (2005). Antiherpetic Activities of Flavonoids against Herpes Simplex Virus Type 1 (HSV-1) and Type 2 (HSV-2) in-vitro. Arch. Pharm.Res.

[48] Nickavar B, Abolhasani L (2013). Bioactivity-Guided Separation of an α-Amylase Inhibitor Flavonoid from Salvia virgata. Iranian J. Pharm. Res.

